# Real-Time Sensor-Embedded Neural Network for Human Activity Recognition

**DOI:** 10.3390/s23198127

**Published:** 2023-09-28

**Authors:** Ali Shakerian, Victor Douet, Amirhossein Shoaraye Nejati, René Landry

**Affiliations:** Department of Electrical Engineering, École de Technologie Supérieure, Montreal, QC H3C 1K3, Canadaamirhossein.shoaraye-nejati@lassena.etsmtl.ca (A.S.N.);

**Keywords:** convolutional neural network (CNN), microcontroller, human activity recognition (HAR), real-time

## Abstract

This article introduces a novel approach to human activity recognition (HAR) by presenting a sensor that utilizes a real-time embedded neural network. The sensor incorporates a low-cost microcontroller and an inertial measurement unit (IMU), which is affixed to the subject’s chest to capture their movements. Through the implementation of a convolutional neural network (CNN) on the microcontroller, the sensor is capable of detecting and predicting the wearer’s activities in real-time, eliminating the need for external processing devices. The article provides a comprehensive description of the sensor and the methodology employed to achieve real-time prediction of subject behaviors. Experimental results demonstrate the accuracy and high inference performance of the proposed solution for real-time embedded activity recognition.

## 1. Introduction

Human activity recognition (HAR) using wearable sensors has demonstrated remarkable achievements through the application of neural networks [1,2,3]. By inputting raw data from accelerometers, gyroscopes, and occasionally magnetometers into pre-trained neural networks, it becomes possible to detect and classify human activities or motion behaviors [4,5]. However, there are notable drawbacks associated with the utilization of wearable sensors. Firstly, real-time inference on neural networks often necessitates an external processing platform, resulting in the transmission of data to a host computer. This reliance on an external device for predictions limits the potential for embedding such systems in compact and lightweight packages for practical real-world applications.

Secondly, the support for microcontrollers in popular machine learning frameworks, such as TensorFlow, has been relatively recent, accompanied by inadequate documentation and limited platform compatibility. Despite these challenges, low-cost sensors, comprising inexpensive microcontrollers and inertial measurement units (IMUs), find extensive use in various applications such as medical and health analysis [6], motion capture [7], and inertial navigation [8]. For human activity recognition, sensors can be placed on different parts of the body to capture data using integrated inertial units. The captured data can be either stored in the sensor’s memory or transmitted to an external device for real-time or post-processing analysis [9,10]. Alternatively, smartphones can directly process measurements from their inertial sensors [11].

Common approaches to processing this data involve traditional machine learning techniques like Support Vector Machines (SVMs) [12] or boosting [13], employing handcrafted features [14], or, in more advanced systems, utilizing neural networks such as multilayer perceptrons (MLP), convolutional neural networks (CNNs), or Long Short-Term Memory networks (LSTMs). Artificial neural networks (ANNs) are gaining popularity due to their state-of-the-art performance, capacity to learn and generalize high-level relationships in data, and their ability to process raw data without the need for handcrafted features. Typically, inference is performed on computers [10] or specialized systems like Nvidia’s Jetson Nano, Google’s Coral USB accelerator, or Intel’s Neural Compute Stick 2, leveraging the processing capabilities of CPUs, GPUs, or TPUs. Inference can also be executed on non-specialized small single-board computers, such as the Raspberry Pi [9].

In a recent study, researchers introduced an innovative wearable multi-sensor data fusion approach that combines supervised machine learning algorithms with multi-resolution time–frequency analysis of received signal strength (RSS) between wearable sensors. Their framework surpasses current state-of-the-art techniques, achieving an impressive maximum classification accuracy of 99.63% [15].

Another study focused on edge fitness and context-aware health monitoring devices, specifically designing a convolutional neural network (CNN)-based human activity recognition (HAR) system. The authors explored the impact of hyperparameters and evaluated the performance of CNN-based methods using acceleration signals from the University of California, Irvine (UCI) HAR benchmark database. They emphasized the significance of selecting appropriate activation functions and segment sizes to optimize accuracy and demonstrated the real-time feasibility of their approach using the Raspberry Pi 4 [16].

To address the challenge of limited labeled data, researchers proposed a CapsLSTM-based model for HAR in smart healthcare [17]. By leveraging accelerometer and gyroscope sensors in smartphones, they incorporated spatio-temporal information to improve recognition. The authors validated their framework on the UCI-HAR and MotionSense databases and achieved comparable test accuracies across different fractions of training data, outperforming other models such as LSTM, 1D-CNN, ConvLSTM, or CNN-LSTM [17].

In a recent survey, researchers conducted an extensive review of Wearable-Based Fall-Related Recognition Systems (WFRSs). These systems are essential for monitoring and ensuring the safety of the elderly, particularly given the rising global aging population. Falls significantly affect the quality of life of older adults, leading to both physical and psychological harm. The United Nations anticipates a surge in the number of individuals aged 65 and older, increasing from 727 million (9.3%) in 2020 to 1.5 billion (16%) by 2050. According to the World Health Organization (WHO), about 28–35% of people aged 65 and over fall each year. These falls result in unintentional injuries, such as fractures and cognitive declines, highlighting the urgency for precise and efficient Fall Recognition Systems (FRSs) [18]. Previous research has examined FRSs, focusing on sensor types and algorithms. Yet, there’s a notable gap in assisting newcomers in choosing the right technology for every phase of their research. To bridge this gap, the paper reviews 48 cutting-edge research articles on WFRSs from three renowned databases. The paper delves into the entirety of WFRSs, spanning data collection to model evaluation, and assesses the advantages and limitations of various sensor counts, data preprocessing methods, feature extraction techniques, and model training approaches [18].

In another study, researchers compared different deep-learning techniques for HAR using ankle inertial signals [19]. They proposed several models based on artificial neural networks and achieved an average classification accuracy of up to 92.8%. Additionally, a lightweight feature extraction technique combined with pruned and quantized CNN was introduced for HAR on wearable devices. By utilizing statistical feature extraction, the authors effectively discriminated between static and dynamic activities. They employed Random Forest (RF) and CNN for specific activity classification, achieving high accuracy while reducing computational and memory consumption [19]. Another research effort explored the use of logistic regression with hyperparameter addition for HAR using smartphone sensors [20]. The authors’ approach yielded improved accuracy results, aiming to enhance the performance of smart devices in daily life.

Additionally, a voting classifier system for HAR that combines multiple machine learning classifiers was introduced [21]. The system utilized classifiers such as logistic regression (LR), K-Nearest Neighbors (KNN), Random Forest (RF), Naive Bayes (NB), and Support Vector Machine (SVM). The authors evaluated the effectiveness of ensemble voting classifiers on the UCI-HAR dataset, with Voting Classifier-II outperforming other classifiers. The integration of deep learning and swarm intelligence optimization algorithms for HAR using wearable sensor data was also investigated [22]. In this study, the authors developed a robust HAR system and proposed a lightweight feature extraction approach based on a residual convolutional network and a recurrent neural network (RCNN-BiGRU). Their feature selection methods, utilizing the marine predator algorithm (MPA), demonstrated superior performance compared to other optimization algorithms.

Furthermore, another study focused on the implementation and evaluation of a real-time HAR system [23]. The researchers explored optimized machine learning techniques for HAR in cyber-physical systems, comparing various algorithms such as Random Forest, Decision Trees, and K-Nearest Neighbors with deep learning algorithms like convolutional neural networks, Long Short-Term Memory, and Gated Recurrent Units. Additionally, they introduced optimization techniques such as Stochastic Gradient Descent (SGD), Adam, and RMSProp [24].

Certain applications require real-time behavior analysis without the possibility of sending data to an external device due to environmental or external constraints. Examples include analyzing athlete performance, detecting soldiers’ behaviors in harsh environments, or studying diver activities underwater. In such scenarios, transmitting data at an adequate rate for external processing may not be feasible, making the adoption of sensor-embedded neural networks a compelling solution. Real-time processing is of paramount importance in these contexts, especially when analyzing on-the-fly sensor data. Convolutional neural networks (CNNs) offer several distinct advantages that make them ideal for this purpose over architectures like LSTM and CapsLSTM. One of the chief benefits is their computational efficiency. Lacking the recurrent connections of LSTMs, CNNs often demand fewer computational resources, which is pivotal for real-time applications where rapid inference is critical. Moreover, CNNs typically possess a lower model complexity when tailored for real-time purposes. They tend to be more streamlined with fewer parameters, facilitating faster model loading and quicker inference times—both essential for real-time sensor data processing. On the other hand, LSTMs, with their intricate recurrent architecture, can often be more memory-intensive and slower in execution, especially when dealing with extensive sequences. Furthermore, CNNs excel in detecting localized patterns that are frequently observed in sensor data. This capability enables immediate recognition of emergent patterns in real-time, avoiding the waiting period associated with processing an entire sequence, a potential shortcoming of certain LSTM configurations. This article delves into real-time human activity recognition using inertial data. By harnessing a CNN and the sensor’s onboard microcontroller, we enable efficient and effective inference, demonstrating the viability and superiority of this approach for real-time, on-device applications.

## 2. Materials and Methods

In this research, a wearable sensor using low-cost MEMS capable of processing inertial data using neural networks on its microcontroller was developed. A mobile application was also created to control and receive information from the sensor. Finally, a 3D-printed case was made to be attached to a user’s chest.

### 2.1. Hardware

In the domain of human activity recognition, sensor selection plays a crucial role in system design. In one study, the researchers employed the ESP8266 sensor (ESP8266-12F), a WiFi-based sensing device, for collecting multi-dimensional received signal strength indicator (RSSI) records [25], where WiFi-based sensing coupled with hierarchical deep learning models achieved recognition accuracies of 96.45% and 94.59% for six different activities in varying environments. In contrast, another study utilizes the 3-DOF accelerometer ADXL345 for motion-based activity recognition, reporting a recognition rate of 99.2% for four key daily activities [26].

**Figure 1:**
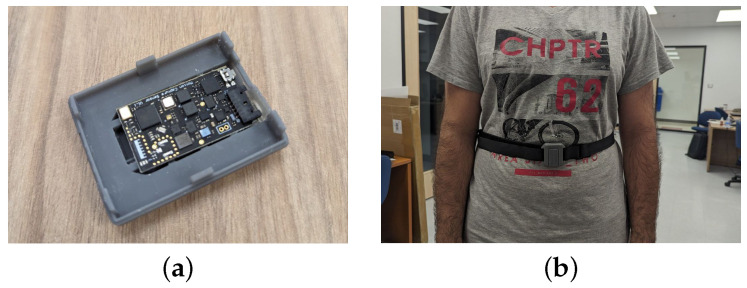
Different views of the ibNav 6.1 sensor. (**a**) The board itself. (**b**) The sensor on the user’s chest.

The sensor outlined in this research incorporates an ICM-20948 9-DOF IMU from InvenSense and an ESP32-WROOM-32 from Expressif Systems, both of which are compatible with the TensorFlow Lite framework for microcontrollers. As depicted in Figure 1a, the sensor’s dimensions are 4.37 cm in length, 1.2 cm in width, and 0.34 cm in height. It can be powered either by a 3.7 V 500 mAh battery or through a USB cable. To contextualize its significance, it is crucial to juxtapose our sensor with other market-available sensors in terms of specifications, price, and SWAP-C. Our study builds upon this foundation while introducing the ibNav 6.1 sensor, which incorporates the ICM-20948. This choice offers several advantages. The ICM-20948 is a versatile and advanced motion sensor module known for its precision in capturing a wide range of motion data, including acceleration and gyroscope measurements. Its integration within the ibNav 6.1 sensor enriches our dataset with additional dimensions of information, facilitating a more comprehensive understanding of human activities in diverse real-world scenarios. By selecting the ibNav 6.1 sensor with the ICM-20948, we aim to leverage these advantages to advance the field of HAR, capitalizing on its enhanced capabilities for a more holistic and accurate recognition of human activities.

Table 1 provides a competitive comparison analysis of sensors designed for HAR. Based on the data presented in this table, the ibNav 6.1 offers superior specifications at a competitive price when compared to its counterparts.

The sensor also provides the capability of transmitting and receiving data wirelessly via the integrated onboard Wi-Fi and Bluetooth features, or, alternatively, through a wired connection. In addition to the ICM-20948 and ESP32, the system necessitated a component with server functionality. This was to facilitate data reception and establish a communicative bridge between the sensor and the end-user. Moreover, this server component was required to possess adequate data storage capacity to accommodate datasets for post-processing, in addition to providing training for the convolutional neural network. The Raspberry Pi 4 Model B was selected to fulfill these needs. A summary of the ICM-20948 specifications is provided in Table 2.

In this study, our sensor equipped with a barometer and magnetometer provided a robust set of sensor data. However, for the specific research focus of human activity recognition (HAR) using CNN and IMU sensors, we made a deliberate choice to exclude the barometer and magnetometer data. This decision was based on the fact that these sensors, although valuable for altitude measurement and orientation estimation, did not directly contribute to our central research objectives related to HAR. To streamline our dataset, reduce dimensionality, and ensure model clarity, we opted to concentrate solely on the IMU sensor data, which is a recognized and widely used source for HAR in the literature.

### 2.2. System Architecture

**Figure 2:**
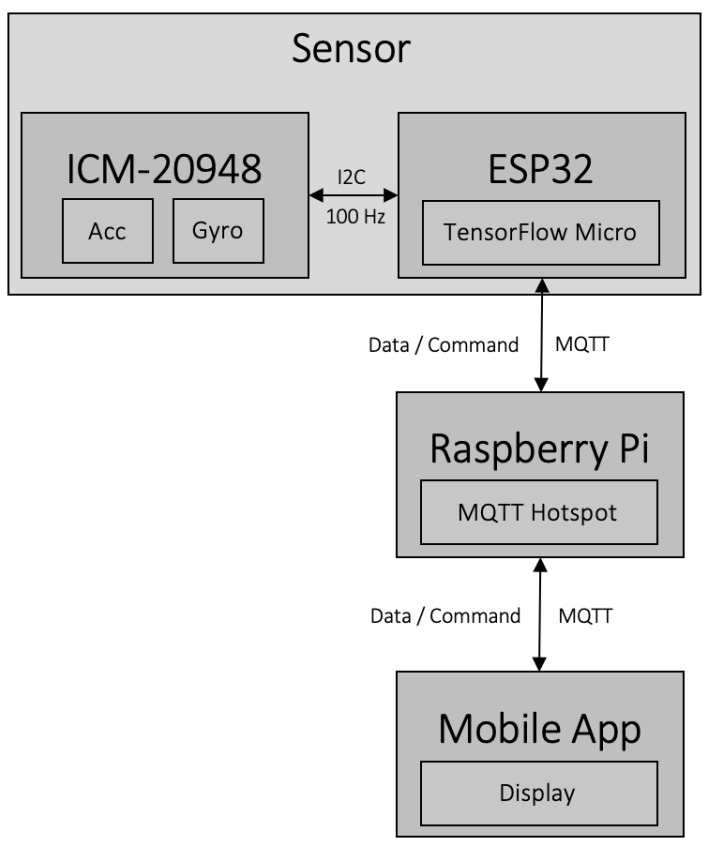
Architecture of the embedded system.

For this system, an iOS and Android application was developed using the Unity Game Engine to control and show real-time measurements from the sensor. Both the application and the sensor connect wirelessly using WiFi to the Raspberry Pi, acting as a hotspot. For communication between components, the lightweight, publish–subscribe messaging protocol MQTT is used. The application can control the sensor by sending simple MQTT commands to it and change, on the fly, various aspects of the sensor such as activate or deactivate the activity prediction task, reset and re-calibrate the IMU, and reboot the device. The application is also used to view the sensor’s prediction of the activities in real-time and various statistics about the system. It is important to note that the module does not require to be connected to the other parts of the system for the HAR task and can function in standalone mode with its integrated battery. Inertial data acquisition is set at a frequency of 100 Hz, those data are then fed to the input of the convolutional neural network embedded into the microcontroller and are also transmitted over MQTT to be stored in the Raspberry Pi memory. The architecture of the system can be seen in Figure 2.

### 2.3. Convolutional Neural Network Model

For this human activity recognition task, a convolutional neural network was chosen due to its state-of-the-art results in activity classification [11] as well as in other tasks and to avoid needing handcrafted features. Additionally, the preference for convolutional neural networks (CNNs) over LSTM and CapsLSTM models for our project was driven by their advantages in simplicity, faster inference times, superior local pattern detection, and efficiency on edge devices, especially with TensorFlow Lite Micro. Additionally, CNNs have a unique strength in learning spatial hierarchies, a feature not as pronounced in LSTMs. The model is composed of three 1D convolution layers with a Rectified Linear Unit (ReLu) as their activation function. Additionally, dropout and max pooling layers are added after each of the convolutions. The output of the last convolution is then flattened and passed to the first dense layer with a ReLu activation and dropout. Finally, a last dense layer is placed at the end of the network with an output shape corresponding to the number of activities to be classified by the softmax activation function. The different layers used in the network are seen in Table 3 and are explained next.

- *1D Convolution:* The convolution layer is used to extract features from data. It applies a convolution operation on its input using a number of *filters* of a set size (*kernel size*) to generate *feature maps*.
- *ReLu:* A Rectified Linear Unit is an activation function. It introduces non-linearity to the network to help learn complex mapping functions. The ReLu activation is defined as follows
  (1) f(x)=max(0,x)
  Other activations exist such as Leaky ReLu, Tanh, Swish, etc.
- *Max pooling:* A pooling layer is used to reduce the dimensionality (*downsampling*) of the feature maps and adds some translation invariance. It reduces the number of parameters and helps prevent overfitting. Max pooling is performed by applying a *max filter* on the feature maps.
- *Dropout:* Dropout layers are a regularization technique to prevent overfitting by stopping neurons to learn specific features of the training data. During training of the network, a neuron is temporarily deactivated (*dropped*) with probability *p*.
- *Dense layer:* A dense or fully connected layer is a regular neural network layer where all neurons in the previous layer are connected to all neurons on the next layer. A fully connected layer expects a 1D vector as input; a flatten layer is used to *flatten* the output of the convolution layers.
- *Softmax:* Softmax is another activation function. It is used for classification tasks by normalizing its input into a probability distribution. Each class to be predicted is assigned a probability. The class with the highest probability is used as the activity predicted by the network.

More information on convolutional neural networks can be found in the paper “Deep Learning” by LeCun et al. [27].

The 3-axis accelerometer (in g) and gyrometer (in rad/s) data from the ICM-20948 IMU are fed at a frequency of 100 Hz, as an input to the network. A timestep of 100 and a 10% sliding window are also used, resulting in an input shape of (None, 100, 6) for the first 1D convolution layer.

Although the model used in this system for the prediction task could have been more complex, the model size is limited due to the 4 MB flash memory of the ESP32-WROOM-32. At the moment, more than 94% of the flash memory is used by the model and the rest of the sensor’s firmware. Adding new layers with new weights to the model would exceed the current available space.

### 2.4. Training and Testing Methodology

To test the activity recognition task using an embedded neural network, we defined six different activities to be classified by the model. Those activities are: walking, jumping, jogging, sitting, crouching, and remaining stationary (meaning no movement of the legs but slight upper-body movements are allowed). The datasets were generated on one user with each dataset corresponding to only one of the six activities. The sensor was placed on the user’s chest, as seen in Figure 1b using an elastic strap-band. A calibration of the IMU is performed upon powering the sensor and the user is then asked to carry out a specific activity. Once the activity is completed, the system is reset and a new activity is performed.

Training and testing datasets were generated separately to avoid introducing any bias in our system. The leave-one-trial-out process described in the paper “Human activity recognition based on wearable sensor data: A standardization of the state-of-the-art” by Jordao et al. [28] was used, except the 10-fold cross-validation process was not applied here because, as mentioned previously, the training and testing datasets were generated at different times.

Discussing the validation approach, the “leave-one-trial-out” method was employed to rigorously assess the model’s robustness across diverse trials. The primary goal was to determine whether a model trained on a specific set of trials could accurately predict activities in trials it had not encountered before. To ensure the integrity and representativeness of the dataset, each trial was meticulously designed to encompass a wide range of activities and scenarios. However, in the realm of real-time systems, the critical concerns of data integrity and potential noise due to network-related challenges take center stage. Addressing these concerns, a meticulous preprocessing and data validation pipeline was implemented. This comprehensive process included meticulous monitoring of time-stamps and sequence integrity, the meticulous handling of missing segments during preprocessing to maintain temporal coherence, and exhaustive testing in an environment with a stable network connection. In cases where data packets encountered transmission delays, a proactive approach involved systematic logging, thorough examination, and the exclusion of any packets deemed corrupted or inauthentic. This stringent data quality control process serves as a cornerstone to guarantee the reliability and precision of results within the context of real-time system applications.

Table 4 shows the distribution of the activities’ datasets between training and testing. Finally, training was performed on 21 epochs using a learning rate of $6\times 10^{-4}$ with the Adam optimizer and a batch size of 32. For real-time testing with the microcontroller, the model was converted into a TensorFlow Lite format. Post-training quantization was used to improve the model size and hardware latency using full integer quantization of weights and activations by supplying a representative dataset of the sensor’s data to the converter. The TensorFlow Lite model was then converted into a TensorFlow Lite FlatBuffer to be integrated into the microcontroller flash memory. During real-time testing, using the sensor, the activities performed by the user were analyzed and matched with the real-time predictions of the convolutional neural network transmitted from the wearable sensor to the application using the MQTT protocol. The accuracy can be calculated with the number of times the user performed an activity and the number of times the sensor predicted the correct performed activity.

## 3. Results and Discussion

This section presents the outcomes of our research, specifically focusing on the results from the training and testing phases of our neural network-based human activity recognition system. Through these results, we aim to elucidate the model’s efficacy under controlled and real-world conditions. A comprehensive discussion following the presentation of each result provides insights into the implications and potential avenues for future research.

### 3.1. ibNav 6.1 Battery Performance Analysis

**Figure 3:**
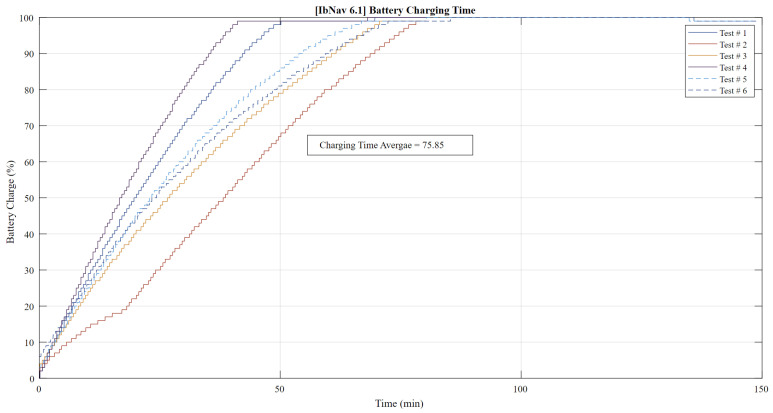
Battery charging time for six different tests.

**Figure 4:**
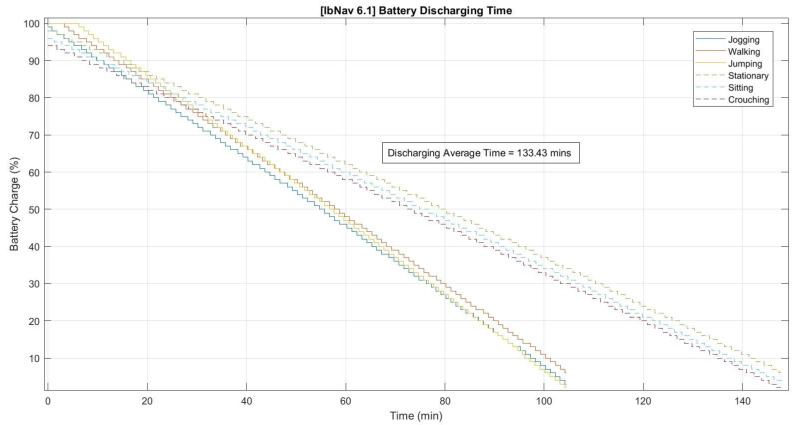
Battery discharging time for six different tests.

In the assessment of the battery performance for ibNav 6.1, several crucial insights were gleaned from its 500mAh battery capacity. As depicted in Figure 3, the charging metrics of the device stood out as particularly noteworthy. The observed charging duration demonstrated both consistency and efficiency, suggesting that the ibNav 6.1 is adeptly designed for minimal downtime, thus enhancing its readiness for swift redeployment after depletion. Shifting the focus to the battery’s discharging behavior, Figure 4 provides a detailed account across six varied tests. These discharge trends furnish a comprehensive understanding of the device’s power consumption across an array of operational scenarios. Notably, the consistency in discharge rates across these tests emphasizes the device’s stability and reliability. This steadiness is particularly valuable when anticipating the device’s performance in prolonged real-time applications. At the same time, any deviations or anomalies in the discharge patterns could be instrumental in identifying specific operational conditions or tasks that may strain the battery, paving the way for potential optimizations in future iterations.

In essence, the battery performance results affirm that the ibNav 6.1 is not just robust in its capacity but also in its operational efficiency, making it a reliable contender for extended real-world deployment scenarios.

### 3.2. Training Results

**Figure 5:**
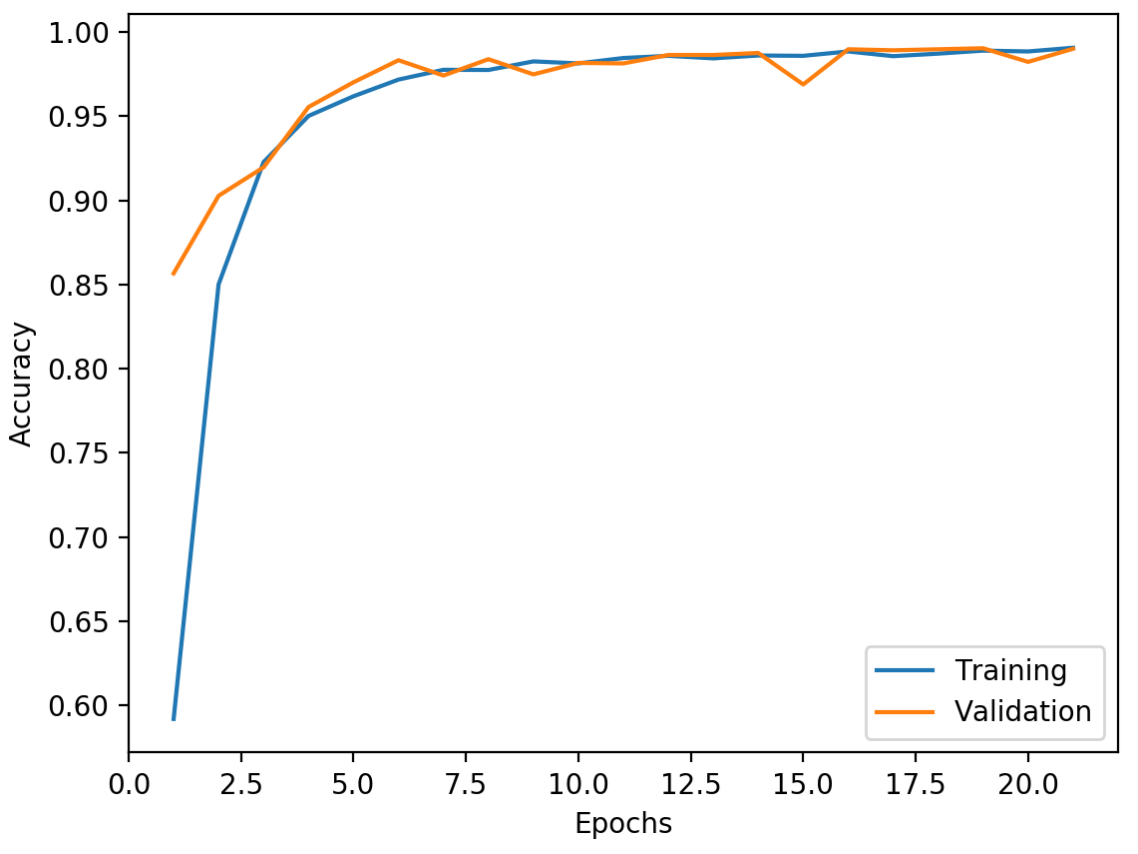
Training and validation accuracy graph.

The primary objective of this project was not to unearth the most advanced neural network for human activity recognition but rather to develop a capability for immediate activity classification, embedded within a microcontroller, thereby eliminating the need for any external computing device. During the training and validation phases, the convolutional neural network illustrated in Figure 5 demonstrated an impressive maximum training accuracy of 99.97% based on a substantial training dataset consisting of 3,630,600 values, inclusive of a 10% overlap. In terms of validation, the figure shows a peak accuracy of 99.47%, leveraging a validation dataset of 1,924,800 values, again with the equivalent overlap. Notably, analogous training results were acquired when employing 2D convolutional layers within the model. Various other models were trialed; however, they were found to surpass the flash memory capacity of the microcontroller, hence proving impractical for our intended use-case.

### 3.3. Testing Results

To evaluate the real-time activity classification capabilities of our system, we initiated a sequence of specific actions for a duration of 10 min, which was undertaken by a test subject. Subsequently, the predictions yielded by the system were relayed to the application, where a comprehensive analysis was performed to distinguish between correct and incorrect predictions. As indicated in Table 5, both the sum of correct predictions and the corresponding accuracy rates for each activity, as well as overall, are tabulated.

It is noteworthy that the embedded convolutional neural network (CNN) successfully executed the human activity recognition classification task with an appreciable degree of accuracy. Indeed, 90.4% of the activities were accurately predicted by the sensor positioned on the test subject’s torso. The misclassified activities are presumably attributable to the limited datasets utilized for training the neural network, which consequently compromised its ability to generalize unseen inertial data across a range of activities.

The enhancement of prediction accuracy is likely to be realized through the creation of larger activity datasets that encompass diverse environmental conditions and scenarios, coupled with an increased participant pool, and retraining the model accordingly.

Additionally, our microcontroller’s inference performance revealed an average processing time of 1.69 s for each inference, given 600 units of inertial data as input. This latency can be mitigated by fully leveraging the dual-core capabilities of the Xtensa microprocessor and optimizing the current model, for instance, by reducing the number of layers and weights in the neural network.

The results obtained in this study demonstrate the successful implementation of a real-time sensor-embedded neural network for human activity recognition (HAR). The system achieved an overall accuracy of 90.4% in predicting various activities, showcasing its potential for practical real-world applications.

The numbers provided in Table 5 represent aggregated counts of specific activities captured across multiple sequences, each lasting 10 min, as mentioned in Section 2. To elaborate further, each 10-min sequence was designed to encompass a mixture of activities. For example, a single sequence could involve a pattern such as 5 instances of jumping followed by 10 of crouching, then 4 of jogging, and so on. By aggregating these specific actions from multiple such 10-min sequences, we arrived at the cumulative counts presented in Table 4. Therefore, the count of 226 for stationary activities represents the total number of stationary instances recorded across all these sequences and similarly for other activities. However, there are several important points to consider and potential areas for improvement that should be discussed.

Firstly, the high training accuracy of 99.97% indicates that the neural network was able to learn and fit the training data effectively. This suggests that the model has sufficient capacity and can capture intricate patterns in the training dataset. However, the validation accuracy of 99.47% was slightly lower than the training accuracy, indicating some degree of overfitting. The model might be too complex for the available data, leading to limited generalization to unseen data. Collecting a larger and more diverse dataset that encompasses various environmental conditions and participant demographics could improve the model’s generalization performance.

Moreover, it is important to acknowledge that the accuracy of the system varied across different activities. While activities like “stationary”, “sitting”, and “crouching” achieved high accuracy, activities such as “walking” and “jumping” had lower accuracies. This discrepancy might be attributed to inherent challenges associated with distinguishing subtle differences in motion patterns between certain activities. Fine-tuning the model or exploring alternative neural network architectures specifically tailored for each activity category could potentially boost accuracy for individual activities.

Furthermore, the limited processing capabilities of the ESP32 microcontroller, with only 4 MB of flash memory, pose restrictions on the complexity of the neural network model that can be used. The model size had to be optimized, which could have affected the overall accuracy. Incorporating external flash memory or exploring specialized hardware accelerators designed for neural networks might enhance both model size and inference speed.

Real-time inference using microcontrollers inherently introduces latency due to the limited computing power compared to dedicated processing units. The average processing time of 1.69 s for each inference indicates a certain level of delay between the actual activity and its prediction. Exploring methods for reducing latency, such as optimizing the network architecture or leveraging hardware acceleration, is crucial to enhance the real-time capabilities of the system.

Reliability in various real-world scenarios is another vital consideration. The system’s performance might be influenced by external factors such as variations in sensor placement, different clothing types, or environmental conditions. Conducting extensive tests in diverse settings and with a larger pool of participants would provide valuable insights into the system’s robustness and practical applicability.

In conclusion, the real-time sensor-embedded neural network for human activity recognition presented in this study represents a significant step towards wearable technologies that can perform HAR without relying on external processing devices. The system’s accuracy and inference performance indicate its potential for numerous applications, including athlete performance analysis, soldier behavior detection, and underwater activity monitoring. However, challenges related to overfitting, activity-specific accuracy, model size, and latency need to be addressed for further improvements. By addressing these issues and conducting more extensive testing, the system could become a valuable tool in a wide range of real-world scenarios, benefiting fields such as healthcare, sports, and security.

## 4. Conclusions

This study presents a wearable sensor embedded with a convolutional neural network, demonstrating considerable proficiency in real-time human activity recognition tasks. Currently, low-cost microcontrollers do not possess the performance capabilities of dedicated computing devices in terms of the complexity of the models that can be utilized. For instance, most real-time vision tasks necessitate intensive processing capabilities beyond the reach of non-specialized, economically efficient microcontrollers.

Nevertheless, the anticipated development of specialized hardware, such as the accelerator module by Coral, is projected to dramatically enhance the efficiency of wearable sensors in implementing neural networks. Future plans involve the design of an advanced board, informed by potential system improvements highlighted in the concluding section of this study. The next stage of development will also focus on creating larger datasets, incorporating multiple participants across diverse environments and encompassing a wider array of activities. The goal is to improve the performance, versatility, and robustness of the system under investigation. Moreover, it is intended to test reinforcement learning methods to enable online training capability.

Lastly, efforts will be made to establish a more robust and precise testing methodology for real-time classification analysis. The proposed improvements are expected to set a solid foundation for advancements in real-time human activity recognition, thus making significant contributions to the broader fields of wearable technologies and artificial intelligence.

A few modifications to the system used in this article could improve accuracy and performance. Currently, the ESP32 microcontroller used in this project is limited to 4 MB of flash memory for stocking both the FlatBuffer array containing the neural network and its weights, and the firmware of the sensor. Developing new boards with external flash memory to increase the total size capacity would allow storing a larger neural network which could improve robustness and outperform the accuracy of the current one. Furthermore, two IMUs with different sensitivity could be used on the same board to improve activity recognition. Nowadays, dedicated computing chips are becoming more available and could be added to the sensor to improve inference capabilities and performance. Reinforcement learning methods could also be used to enable online training of the model to increase accuracy over time. Finally, a few case studies could be carried out to select the best part of the body to place the sensor, for example the waist or the hip rather than on the chest.

## Figures and Tables

**Table 1:** Comparison of sensors.

	MetaMotionC	MetaTracker	MetaMotionR	Notch	IbNav 6.1
Manufacturer	mbientlab	mbientlab	mbientlab	notch	Lassena
Price	$81.99	$60.99	$86.99	$63.16	$50
Battery	200 mAh	600 mAh	100 mAh	70 mAh	500 mAh
Memory	8 MB	4 MB	8 MB		4 MB + SdCard
Radio	BLE	BLE	BLE	BLE	BLE/BT + WiFi
Real-Time Sampling	100 Hz		100 Hz	100 Hz	100 Hz
Accelerometer	X	X	X	X	2X
Gyroscope	X	X	X	X	2X
Magnetometer	X		X	X	2X
Temperature	X	X	X		X
Barometer	X	X	X		X
Humidity		X			X
Sensor Fusion	X		X		X
Enclosure	IP40	IP54	IP40	IP67	IP67
Weight	0.2 oz	0.7 oz	0.2 oz		
Case Dimensions	25 mm diameter × 4 mm	52 mm × 35 mm × 15 mm	27 mm × 27 mm × 4 mm		43.75 mm × 34 mm × 12.2 mm

**Table 2:** Specifications of the ICM-20948.

Specifications	Accelerometer	Gyroscope	Magnetometer	Barometer
Measurement Range	±2 g, ±4 g, ±8 g, ±16 g	±250 dps, ±500 dps, ±1000 dps, ±2000 dps	±4900 μT	260–1260 hPa
Sensitivity	16,384 LSB/g	131 LSB/dps	0.15 μT/LSB	0.01 hPa/LSB
Noise Density	200 μg/$\sqrt{\mathrm{Hz}}$	20 mdps/$\sqrt{\mathrm{Hz}}$	0.15 μT/$\sqrt{\mathrm{Hz}}$	0.01 hPa/$\sqrt{\mathrm{Hz}}$
Output Data Rate (ODR)	4 Hz–1.12 kHz	4 Hz–8 kHz	4–100 Hz	1–200 Hz
Current Consumption (Operating)	450 μA	1.2 mA	90 μA	0.9 mA

**Table 3:** Summary of the CNN model.

Layer (Type)	Output Shape	Parameter #
conv1d_1	(None, 99, 128)	1664
max_pooling1d_1	(None, 49, 128)	0
dropout_1	(None, 49, 128)	0
conv1d_2	(None, 48, 128)	32,896
max_pooling1d_2	(None, 24, 128)	0
dropout_2	(None, 24, 128)	0
conv1d_3	(None, 23, 128)	32,896
max_pooling1d_3	(None, 11, 128)	0
dropout_3	(None, 11, 128)	0
flatten_1	(None, 1408)	0
dense_1	(None, 128)	180,352
dropout_4	(None, 128)	0
dense_2	(None, 6)	774

**Table 4:** Training and validation dataset repartition.

Activity	Train Dataset (min)	Validation Dataset (min)
Stationary	20	20
Jumping	5	2
Walking	39	13
Jogging	20	10
Sitting	9	2
Crouching	4	2
Total	97	49

**Table 5:** Number of accurately predicted activities during testing.

Activity	Number of Predictions	Correct Predictions	Accuracy
Stationary	226	222	98.2%
Jogging	220	214	97.3%
Walking	248	209	84.3%
Jumping	213	179	84%
Sitting	211	189	89.6%
Crouching	208	186	89.4%
Total	1326	1199	90.4%

## Data Availability

Not applicable.
